# Cutaneous Manifestation of Metastatic Infantile Choriocarcinoma

**DOI:** 10.1155/2014/104652

**Published:** 2014-08-12

**Authors:** Timothy Brooks, Laura Nolting

**Affiliations:** Department of Emergency Medicine, Palmetto Health Richland, Suite 350, 14 Medical Park, Columbia, SC 29203, USA

## Abstract

Infantile choriocarcinoma is a highly malignant rare germ cell tumor that arises from the placenta. Simultaneous intraplacental choriocarcinoma involving both mother and infant is extremely rare. Cutaneous metastasis in infantile choriocarcinoma is even rarer with only a few case reports available. Here we describe a case of a female neonate who presented to the ED with a rapidly growing and bleeding vascular lesion to her right cheek. She was eventually diagnosed by biopsy with metastatic choriocarcinoma. In addition to the cutaneous tumor, she also had metastatic disease in her lungs. Her mother was subsequently found to have choriocarcinoma with metastatic disease to the lungs as well.

## 1. Introduction

Choriocarcinoma is an aggressive malignant tumor of placental trophoblastic cells. Reports of the disease are presented in the literature as case reports and review articles. Metastatic spread to the fetus is rare, and diagnosis is often difficult and delayed. In this case report, we describe the clinical, laboratory, and radiographic findings of a neonate presenting to the children's emergency department who was ultimately diagnosed with metastatic choriocarcinoma.

## 2. Case

**Figure 1:**
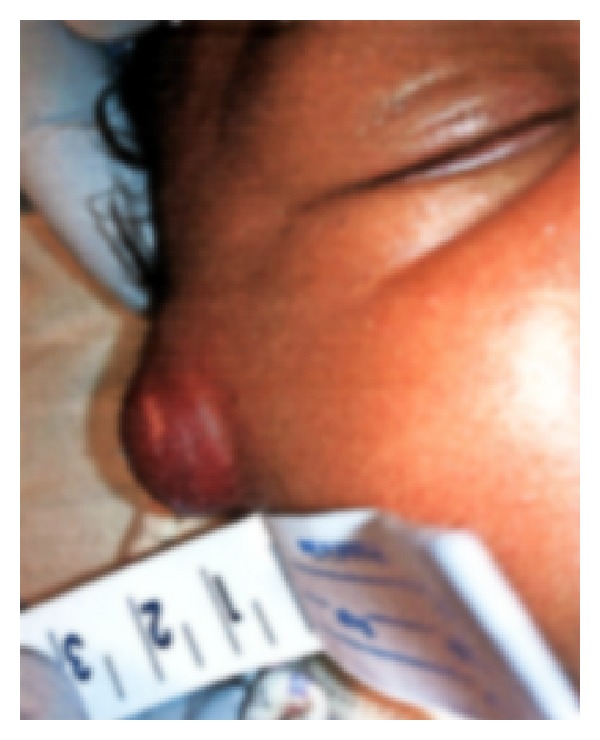
Facial lesion.

**Figure 2:**
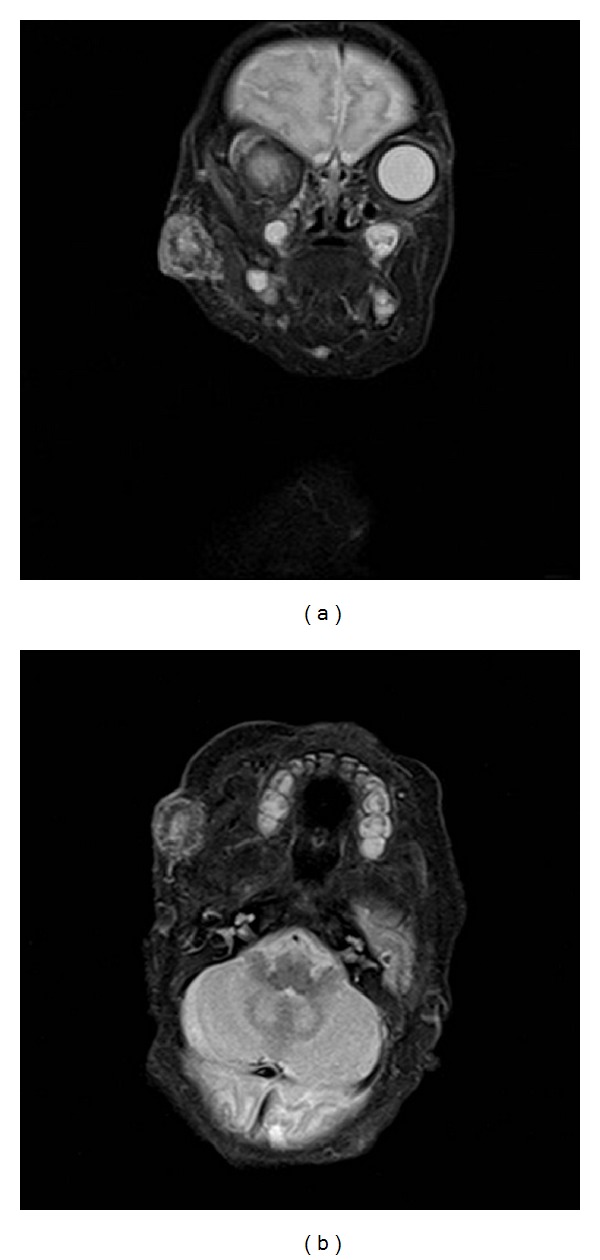
(a) Coronal MRI at 11 days. (b) Axial MRI at 11 days.

The infant presented to the ED the first time on the 11th day of life for evaluation of a right-sided facial mass because the mother felt it was getting bigger and darker. At the time of birth, she was noted to have a 2 cm subcutaneous mass on the right cheek (Figure 1). The lesion was dark red in color and blanched with pressure. It was firm and there was no evidence of fluctuance or surrounding cellulitis. It did not appear to be painful on palpation. It measured 2 × 2 cm in the ED. The infant had no medical history and was born at 35 weeks via spontaneous vaginal delivery, and the pregnancy was only complicated by the mother having* Trichomonas vaginalis* that was treated with metronidazole during her third trimester. An MRI was performed and interpreted as a benign vascular lesion such as a hemangioma. It measured 17 × 18 × 19 mm with a necrotic center. There was no evidence of invasion to adjacent facial structures (Figures 2(a) and 2(b)).

Her CBC, PT, PTT, INR, and fibrinogen were all within acceptable limits.

She was subsequently admitted to the PICU for propranolol therapy to treat the hemangioma. The mother noted that the lesion had decreased in size and color intensity within 18 hours of initiation of propranolol. The child was discharged to follow-up as an outpatient.

On day 18 of life she underwent incision and drainage, without a histological sample, by ENT for what was thought at that time to be an involuted hemangioma. She had done well postoperatively until 12 days later when the lesion began to bleed prompting the mother to bring her to the ED. Vitals were stable and laboratory values were within acceptable limits. The ED physician, who examined the patient in the ED, consulted ENT who felt the infant was stable for discharge after starting her on oral prednisolone and continued the propranolol.

She returned to the ED 8 days later, on day 37 of life, with an enlarging mass and significant bleeding. The lesion had increased in size despite continuation of steroid and propranolol therapy. The mother denied any complicating symptoms including fever, cough, congestion, increased fussiness or irritability, feeding intolerance, or change in bowel or bladder habits.

In the ED, temperature was 97.7 rectally, pulse 159, respiratory rate 44, blood pressure 67/37, and pulse ox 97%. On exam the child had a large erythematous right facial lesion with an involuting necrotic center now measuring 4 × 4 cm. There was a significant amount of clot burden stranding from the lesion. The child had appropriate behavior and neurologic findings and the remainder of the exam was normal.

There were significant normocytic anemia and leukocytosis found on CBC. Hemoglobin (MCV), RBC count, hematocrit, and platelets were 7.4 g/dL (90.3), 2.36 M/uL, 21.3%, and 297, respectively. The peripheral leukocyte count was 20,400/uL with 28% polymorphonuclear leukocytes, 59% lymphocytes, and 10% monocytes. Nineteen days prior to the hemoglobin, RBC count, hematocrit, and leukocyte count were 14.1 g/dL, 4.12 M/uL, 38.8%, and 12,300/uL, respectively.

**Figure 3:**
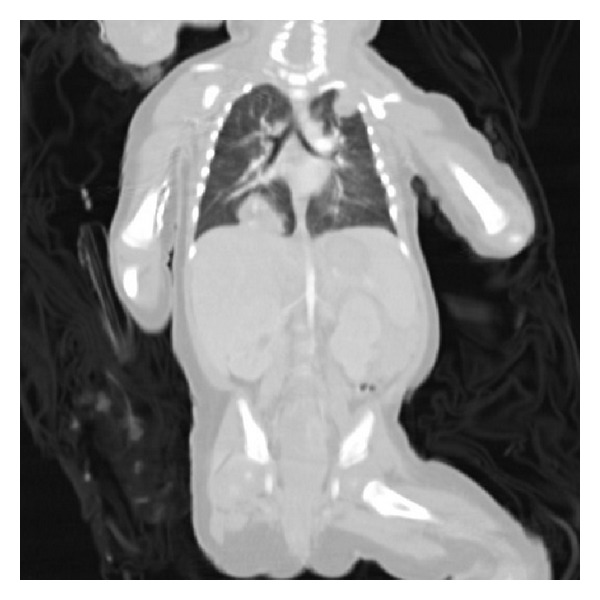
CT of the chest with metastasis.

The child was emergently transfused with packed red cells and transferred to another hospital for embolization where she was ultimately diagnosed with choriocarcinoma by biopsy. She returned to our facility and began chemotherapy, which included bleomycin, etoposide, and cisplatin. Bilateral pulmonary nodules were found on CT scan (Figure 3). There were no other areas of metastasis.

Only after the diagnosis was made histologically and chemotherapy initiated in the child was the *β*-hCG level checked. Subsequently, the infant's mother was evaluated and found to have metastatic disease as well and underwent chemotherapy. Both mother and child are doing well with resolving metastasis and negative *β*-hCG levels.

## 3. Discussion

Choriocarcinoma is a rare malignant disease that arises from the trophoblastic cells of the placenta. It is characterized by secretion of human chorionic gonadotropin (*β*-hCG). Maternal choriocarcinoma is extremely rare, occurring in an estimated 1 in 50,000 live births [1]. About 50 percent of cases of choriocarcinoma arise from complete hydatidiform mole, an additional 25 percent arise after normal pregnancies, and 25 percent follow spontaneous abortion or ectopic pregnancy [2].

Infantile choriocarcinoma is even rarer. Less than 30 cases have been described in the literature [3]. Newborn infants tend to present with a characteristic clinical picture of anemia, hepatomegaly, and precocious puberty [4]. Infantile choriocarcinoma occurs between 0 and 6 months of age. In our case, our patient was born with metastatic disease but presented with only the cutaneous manifestation making it even more difficult to diagnose. Metastasis is common and usually affects liver, lung, brain, and skin, in that order [5]. Infantile cutaneous manifestation of the disease is extremely rare. Of 208 neonates between 1955 and 2010 with malignancies and cutaneous metastases, only 4 patients (1.9%) had cutaneous metastasis due to choriocarcinoma and it was generally associated with poor prognosis [6]. Approximately one-quarter of infants with choriocarcinoma present with symptoms at birth and diagnosis can be easily confirmed with serum *β*-hCG [7]. These tumors are highly vascular and friable, so biopsy may be difficult and even dangerous [8]. Anemia may be gradual or may result from tumor rupture. Transfusion, as in our case, may be required. Failure to thrive is nonspecific and may only manifest as feeding difficulties [9]. Hemoptysis and respiratory failure may be the primary manifestations of lung involvement. Brain and skin involvement are rarely seen and usually indicated advanced disease [5].

Choriocarcinoma is a very aggressive malignancy and death may result from delays in diagnosis. Therefore, early intervention is critical for limiting the progression of disease. In infants, without appropriate treatment, death usually occurs within 3 weeks of initial presentation [8]. Fortunately, despite its aggressive nature, this cancer responds very well to chemotherapeutic agents, even in the presence of widespread metastases [9]. The chance of long-term survival, even in patients with cerebral metastases at presentation, is approximately 80% [7]. Both patients described responded very well to chemotherapy, without significant complications, and currently have shown no progression or recurrence of disease, as indicated by negative *β*-hCG levels, in the two years since diagnosis. Had a *β*-hCG level, which is universally positive in infantile choriocarcinoma and excludes the diagnosis if negative, been known early in the clinical course, chemotherapy could have been initiated sooner [4].

Because a history of maternal choriocarcinoma is associated with a risk of infantile choriocarcinoma in subsequent pregnancies, current guidelines suggest that women with such a history should be checked for *β*-hCG at 6 and 10 weeks following a subsequent pregnancy, regardless of the outcome [4, 7].
